# Impact of industrial production system parameters on chicken microbiomes: mechanisms to improve performance and reduce *Campylobacter*

**DOI:** 10.1186/s40168-020-00908-8

**Published:** 2020-09-09

**Authors:** Aaron McKenna, Umer Zeeshan Ijaz, Carmel Kelly, Mark Linton, William T. Sloan, Brian D. Green, Ursula Lavery, Nick Dorrell, Brendan W. Wren, Anne Richmond, Nicolae Corcionivoschi, Ozan Gundogdu

**Affiliations:** 1 Moy Park, 39 Seagoe Industrial Estate, Portadown, Craigavon, Co. Armagh BT63 5QE UK; 2 School of Engineering, University of Glasgow, Glasgow, G12 8LT UK; 3 Food Microbiology Unit, Agri-Food and Biosciences Institute, Newforge Lane, Belfast, BT9 5PX UK; 4 Institute for Global Food Security, School of Biological Sciences, Queen’s University Belfast, Biological Sciences Building, Belfast, BT9 5DL Northern Ireland; 5 Faculty of Infectious & Tropical Diseases, London School of Hygiene and Tropical Medicine, Keppel Street, London, WC1E 7HT UK

**Keywords:** Chicken, Microbiome, *Campylobacter*, Production systems, Environmental filtering, Phylogenetic signal, Competitive exclusion, Diversity

## Abstract

**Background:**

The factors affecting host-pathogen ecology in terms of the microbiome remain poorly studied. Chickens are a key source of protein with gut health heavily dependent on the complex microbiome which has key roles in nutrient assimilation and vitamin and amino acid biosynthesis. The chicken gut microbiome may be influenced by extrinsic production system parameters such as *Placement Birds/m*^*2*^ (stocking density), feed type and additives. Such parameters, in addition to on-farm biosecurity may influence performance and also pathogenic bacterial numbers such as *Campylobacter*. In this study, three different production systems ‘Normal’ (N), ‘Higher Welfare’ (HW) and ‘Omega-3 Higher Welfare’ (O) were investigated in an industrial farm environment at day 7 and day 30 with a range of extrinsic parameters correlating performance with microbial dynamics and *Campylobacter* presence.

**Results:**

Our data identified production system N as significantly dissimilar from production systems HW and O when comparing the prevalence of genera. An increase in *Placement Birds/m*^*2*^ density led to a decrease in environmental pressure influencing the microbial community structure. Prevalence of genera, such as *Eisenbergiella* within HW and O, and likewise *Alistipes* within N were representative. These genera have roles directly relating to energy metabolism, amino acid, nucleotide and short chain fatty acid (SCFA) utilisation. Thus, an association exists between consistent and differentiating parameters of the production systems that affect feed utilisation, leading to competitive exclusion of genera based on competition for nutrients and other factors. *Campylobacter* was identified within specific production system and presence was linked with the increased diversity and increased environmental pressure on microbial community structure. Addition of Omega-3 though did alter prevalence of specific genera, in our analysis did not differentiate itself from HW production system. However, Omega-3 was linked with a positive impact on weight gain.

**Conclusions:**

Overall, our results show that microbial communities in different industrial production systems are deterministic in elucidating the underlying biological confounders, and these recommendations are transferable to farm practices and diet manipulation leading to improved performance and better intervention strategies against *Campylobacter* within the food chain.

Video Abstract

## Background

Chickens are a key source of protein for humans where poultry production is predicted to produce approximately 130 million tons of chicken meat in 2020 [1, 2]. Sustainable poultry practices are needed to help maintain an adequate supply of poultry products for the increasing human population without compromising the chicken or human health [3]. Selective breeding programmes has resulted in chickens that efficiently convert food into body mass, as defined by feed conversion ratios (FCR) [4]. The fundamental component needed to ensure an efficient poultry production is highly dependent in having an optimised nutrition and production setup [1].

Production systems vary immensely between countries, businesses and at farm level. Certain parameters such as *Placement Birds/m*^*2*^ (stocking density), *Protein_perc_ration* (protein percentage within ration) and *Energy_of_ration* (energy content) in relation to the feed are key determinants that if varied, may directly influence chicken microbial community structure, thus impacting performance and potentially reduction of pathogenic bacteria. Chicken diets are typically formulated to enhance production efficiency. However, some diets are formulated to enhance human health, such as diets containing Omega-3 polyunsaturated fatty acids (PUFA’s) [5]. There is anecdotal evidence at farm level suggesting that this enrichment has potential to improve chicken gut health and performance or reduce pathogen colonisation.

Many bacterial species found within the microbiome of farmed animals can potentially be considered pathogens and detrimental to human health. One of these pathogens is *Campylobacter* which is the leading cause of human foodborne bacterial gastroenteritis causing bloody diarrhoea, fever and abdominal pains in humans and can also cause post infectious sequelae such as Guillain-Barré syndrome which is a potentially fatal paralytic autoimmune illness [6]. *Campylobacter jejuni* (predominant species causing infection to humans) colonises chicken caeca with relatively high numbers (> 10^9^ CFU/g) and can be pathogenic to the chicken, with this dependent on the genetics of host and strain of infection [7–10]. Although there exist many intervention studies, in an industrial farm environment, we currently do not understand why we typically see *Campylobacter* at approximately two weeks into the chicken life cycle [11–13]. The natural growth and flux of the gut microbiome may have a role to play [14]. This is further convoluted by the fact that there are very few studies on how chicken diet impacts *Campylobacter* presence within the chicken gut microbiome, particularly from within an industrial farm environment.

Chicken performance and gut health is heavily dependent on the complex gut microbial community which plays a role in nutrient assimilation, vitamin and amino acid biosynthesis and prevention of pathogen colonisation [15–18]. The microbiota is responsible for hydrolysing indigestible carbohydrates and polysaccharides allowing further fermentation by other members of the gut ecosystem that produce short chain fatty acid (SCFA), in turn allowing utilisation by the host [1]. The relationship between gut microbiota, chicken health and performance represents a tripartite that has been under the scrutiny of the research community with the prospect of improving the efficiency of current microbiome manipulation strategies [19, 20]. As an example, a xylanase gene from chicken caecum has been isolated and overexpressed, potentially leading to development of new feed additives for industrial application [21]. Our understanding of diet and its impact on the intestinal microbiota is still nascent and requires further exploration.

In the present study, we aim to build on our previous research where we performed a comprehensive analysis of the chicken caecal microbiome from days 3 to 35 investigating the driving forces of bacterial dynamics over time, and how this relates to *Campylobacter* appearance within a natural habitat setting [14]. Microbial variation over time was heavily influenced by the diet, where significant shifts in bacterial composition were observed [14]. The factors affecting host-pathogen ecology in terms of microbial community structure remain poorly studied at an industrial farm level. Therefore, extending our previous work and in view of the above ambitions, in this study, three different industrial production systems, namely, ‘Normal’ (N), ‘Higher Welfare’ (HW) and ‘Omega-3 Higher Welfare’ (O) were investigated at day 7 and day 30, along with extrinsic parameters (which were not available in our previous study) to ascertain mechanisms on improving the overall performance of chickens and also elucidating the role of microbial community dynamics on revealing *Campylobacter*.

## Materials and methods

### Ethics statement

Euthanasia of birds was carried out as previously described [14] under veterinary supervision alongside routine veterinary diagnostic inspection and after consultation and approval from the ethical committee within Moy Park. Briefly, euthanasia was performed either by dislocation of the neck or by dislocation of the neck followed by anaesthesia with isoflurane depending on the weight of the bird.

### Experimental design, broilers and sample collection

Chickens reared under three different industrial growing regimes, ‘Normal’ (N), ‘Higher Welfare’ (HW) and ‘Omega-3 Higher Welfare’ (O), were sourced from three different contract farms supplying chicken to Moy Park (39 Seagoe Industrial Estate, Portadown, Craigavon, Co. Armagh, BT63 5QE, UK).

Although the three production systems differ in receiving chicks from multiple flocks, our analysis suggested that they had no bearing on microbial community structure at finer level (Fig. 3 and discussion later) although some parameters were implicated as significant in PERMANOVA analysis. All chicks were Ross 308 as hatched (AH) and were supplied from the same Moy Park hatchery to all three farms on 11 October 2018. All birds were grown in typical industrial poultry houses and were raised on a four-stage diet made up of a starter, grower, finisher and withdrawal ration; however, composition of these diets differed across the three growing regimes. For farm N, birds were offered standard starter ration from days 0 to 11, standard grower ration from days 11 to 22 and standard finisher from day 22 to day 34 before moving to a standard withdrawal ration prior to slaughter. For farm HW, birds were offered higher welfare starter ration from days 0 to 11, higher welfare grower ration from days 11 to 23 and higher welfare finisher from day 23 to day 31 before moving to a higher welfare withdrawal ration prior to slaughter. For farm O, birds were offered higher welfare starter ration from days 0 to 11, higher welfare grower ration from days 11 to 20 and Omega-3 finisher ration from day 20 to day 30 before moving to an Omega-3 withdrawal ration prior to slaughter. Both Omega-3 finisher rations are identical to HW equivalent ration, but with addition of an Omega-3 premix produced by Devenish Nutrition Ltd (Belfast, UK) and added at the feed mill. At day 7, 19 chickens and at day 30, 10 chickens were randomly removed from the same single poultry house on each of the three farms.

### *Campylobacter* isolation and identification

The contents of a pair of caeca were transferred into a sterile stomacher bag (~ 10 g ± 1 g), diluted with maximum recovery diluent (MRD) (Oxoid, UK) buffer to make a 1/10 dilution and stomached at 260 rpm for 1 min. Further decimal dilutions were carried out in MRD to give a range of dilutions suitable to achieve countable plates. One hundred micro litres of the original suspension (10^−1^ dilution) was inoculated onto duplicate plates of modified charcoal cefoperazone deoycholate agar (mCCDA) (Oxoid, UK). The inoculum was spread uniformly over the surface of the agar plate with a sterile spreader until fully absorbed. This spread plating was repeated for all the other decimal dilutions. When low numbers of *Campylobacter* were expected, the limit of detection was increased by also spread plating 1 ml of the original suspension. The 1-ml inoculum was inoculated over three plates. The plates were incubated in a microaerophilic atmosphere (5% oxygen, 10% carbon dioxide and 85% nitrogen) at 41.5 °C and examined after 44 ± 4 h for typical colonies of *Campylobacter* spp. Plates containing less than 300 colonies were counted. Colonies were considered typical if they were greyish (often with a metallic sheen), flat and moist, with a tendency to spread. *Campylobacter* numbers were expressed as CFU/g caecal content. Genomic DNA (gDNA) was also extracted from chicken caeca for 16S metagenomics experiments.

### Poultry growth and performance measurements

Performance parameters on per flock basis were recorded in line with typical industrial practices. *Weight_Gain_per_Day*, *Feed_Conversion_Ratio*, *Total_Mortality_percentage*, *Energy_of_Ration*, *Protein_perc_ration*, *Water_Consumption_per_Bird*, *Age_At_Thin*, *Age_at_Total_Depopulation*, *PMI_Rejects_percentage*, *Hockmark_percentage*, *Pododermatitis_percentage*, log_CFU_per_g_Campylobacter and *European Poultry Efficiency Factor* (*EPEF*) were all captured (Supplementary S13). These variables were then correlated with the microbial community’s composition in various statistical analyses.

### DNA extraction, 16S rRNA amplification and sequencing

Caecal gDNA was extracted using QIAamp DNA Stool Mini Kit according to the manufacturer’s instructions and stored at − 20 °C. 16S metagenomic sequencing library construction was performed using Illumina guidelines (Illumina, USA). The 16S ribosomal primers used were V3 (tcgtcggcagcgtcagatgtgtataagagacagcctacgggnggcwgcag) and V4 (gtctcgtgggctcggagatgtgtataagagacaggactachvgggtatctaatcc) [22, 23]. A second PCR step was performed based on the manufacturer’s guidelines to attach dual indices and Illumina sequencing adapters using the Nextera XT Index kit. Sequencing was performed on Illumina MiSeq at LSHTM using a v3 300 bp paired-end kit.

### Bioinformatics and statistical analysis

Details are described in the supplementary methods (Supplementary S12).

## Results

### Diversity patterns representative of the production systems

**Fig. 1:**
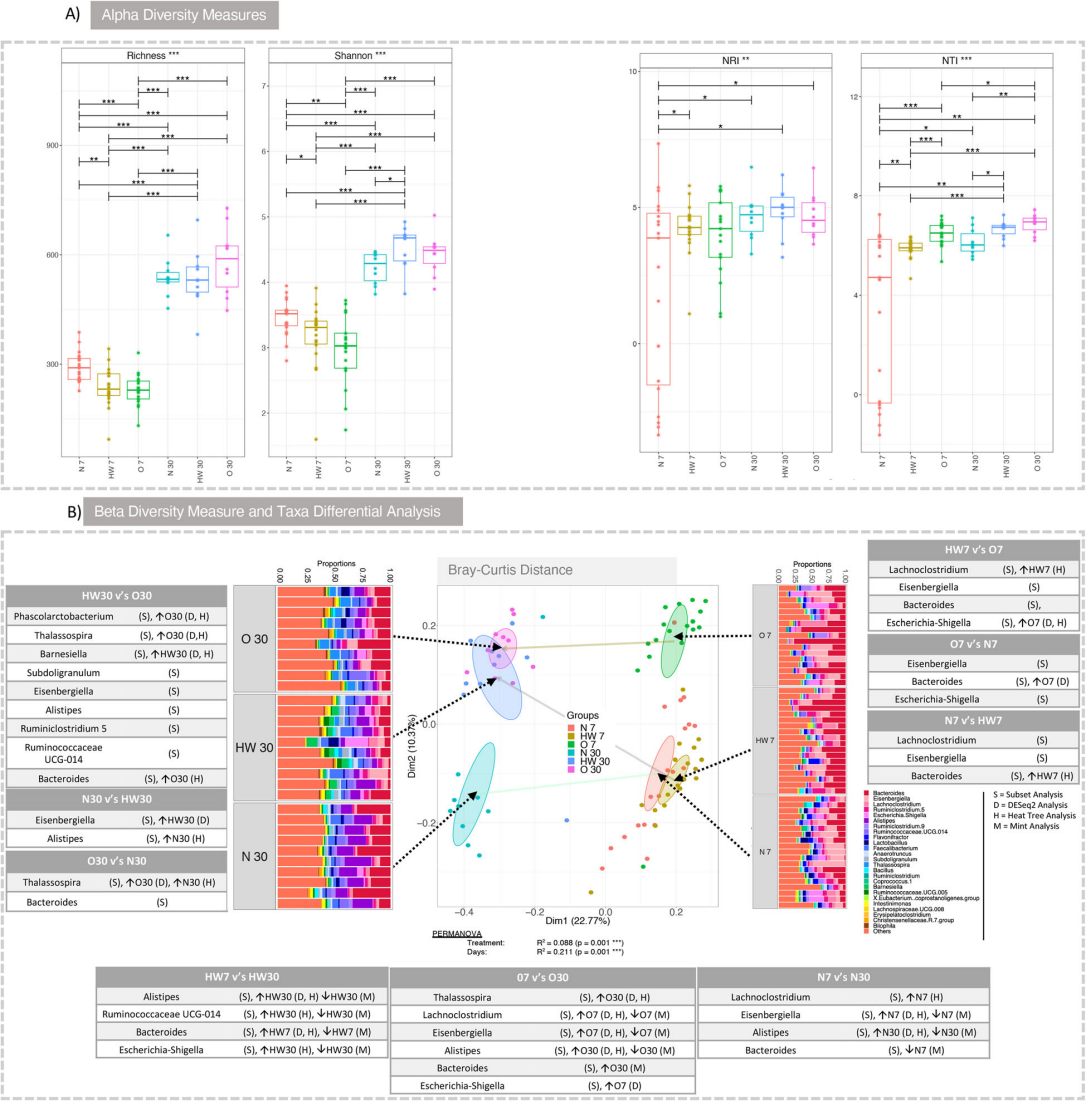
Microbial diversity and community structure. **a** Alpha diversity (Richness and Shannon entropy) and environmental filtering (NRI/NTI) measures respectively. Lines (**a**) connect two categories where the differences were significant (ANOVA) with *P < 0.05, **P < 0.01, or ***p < 0.001. **b** Beta diversity using Bray-Curtis distance measure along with top-25 genera observed in all samples grouped by categories. The tables represent taxa that were found to be significant based on subset analysis (Supplementary S1), i.e. those genera selected in the subsets that explain roughly the same distance between samples as all the genera. Additionally, if the taxa were found to be differentially expressed based on other analyses, such as DESeq2 (Supplementary S11), MINT (Supplementary S2) and differential heat tree (Fig. 2), the categories they up- and downregulated are represented with corresponding up and down arrows. For example, in HW30 vs O30 comparison, ‘(S), O30 (D, H)’ for *Phascolarctobacterium* should be read as selected in subset analysis: (S) and upregulated in O30 according to both DESeq2 and Differential Tree: O30 (D, H)

At alpha-diversity level, to investigate how diversity within the samples were influenced by the production system, both richness and Shannon entropy were calculated for day 7 and day 30 samples from production systems N, HW and O. In the host microbiome, time has a significant effect on microbial richness with all production systems significantly increasing from day 7 to day 30 (Fig. 1a). For day 7, production system N displayed the highest microbial richness when compared to HW and O. At day 30, no statistical difference in terms of richness was identified between the three production systems. Beta diversity using *Bray-Curtis* was measured, along with these operational taxonomic units (OTUs) collated together at genera level with only top-25 most abundant genera shown next to the PCoA diagram (Fig. 1b). At day 7, in terms of abundance counts, samples from production system O are far off from the clusters that contain samples from N and HW, respectively. On the other hand, at day 30, N seems to have a different community structure as compared to N and HW production systems, respectively. The breakdowns of taxa going from finer (OTUs), up coarser levels (family, class, phylum, etc.), are shown in Supplementary S3. Note, we are only considering the top-25 most abundant taxa identified at different taxonomic levels. Of interest, clear differences were observed using visual cues when comparing production system N at day 30 to HW and O, where genera *Bacteroides* and *Alistipes* were present in production system N at higher abundances.

Ecological drivers of microbial community were explored observing the clustering in the phylogenetic tree of the OTUs and utilising phylogenetic alpha diversity measures such as NRI/NTI. Positive NRI/NTI values indicate strong phylogenetic clustering (driven by environmental filtering), whereas reduced values represent phylogenetic overdispersion (environment has little or no role to play). Here, using NRI and NTI, we can observe an increase in N, HW and O samples respectively from day 7 to day 30. Since chicken caeca are already a constrained environment to begin with (as opposed to real environmental datasets), the values < 0^1^ (traditionally this implies stochasticity) may not be feasible to ascertain randomness/stochasticity/competitive exclusion principle, and hence values should be taken relatively with an increasing value implying increasing host environmental pressure (in the immediate case it is the environment within a chicken, whilst extrinsic environmental factors such as surroundings where chickens are confined and their diet may also have a role to play). Figure 1a corroborates our findings from our previous longitudinal study [14].

### Key drivers of microbial community structure variation in terms of beta diversity

**Fig. 2:**
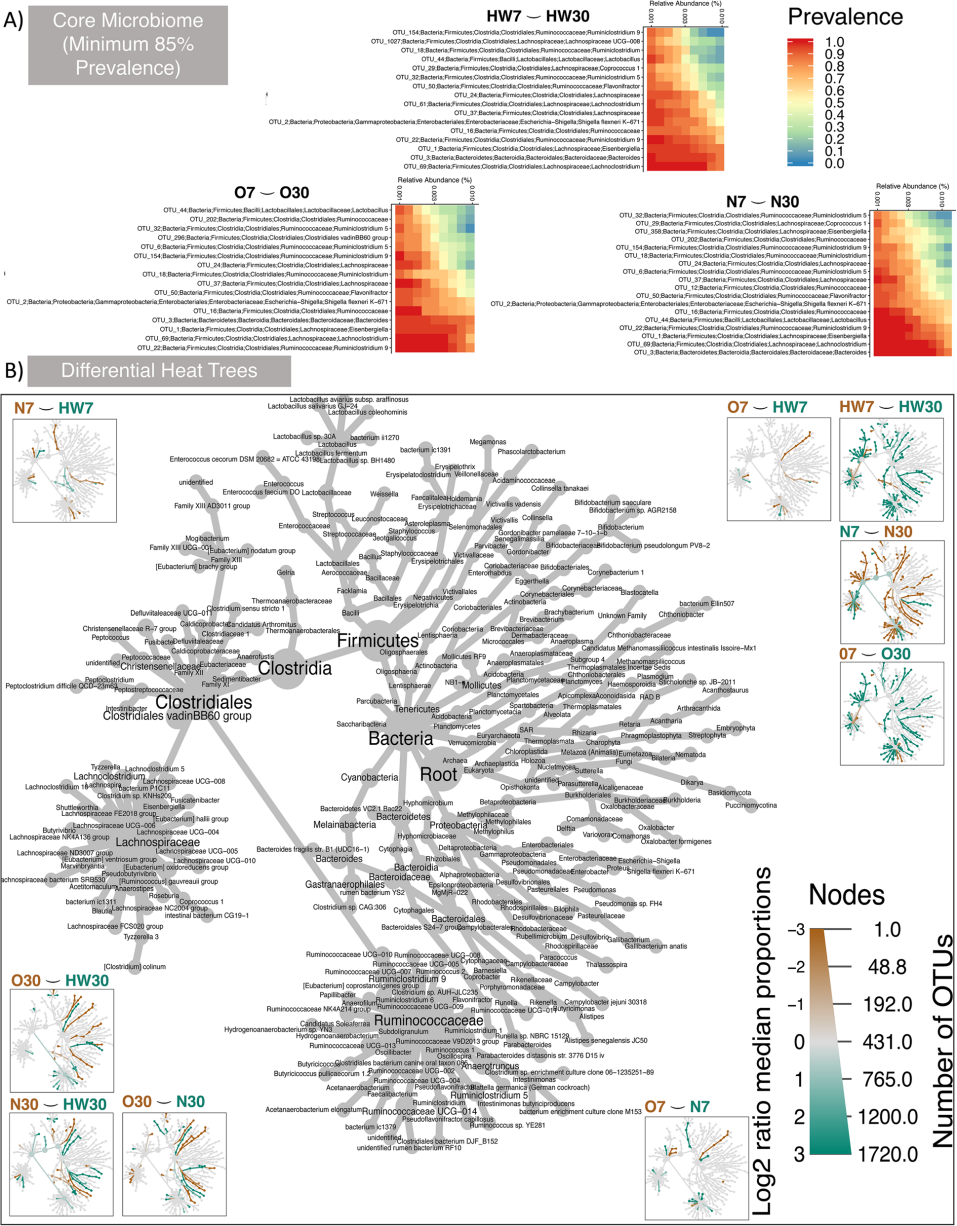
Taxa that persist and those that are differentially abundant. **a** Core microbiome analyses that persist in 85% of the samples for different production systems (day 7 and day 30). In the heat maps, the OTUs are sorted by their abundances with those on the top being low abundant, whereas those at the bottom are highly abundant. **b** Differential heat tree with taxonomy key given in the middle, and the branches where they are upregulated are coloured according to their respective categories shown on top of each subpanel

Next, we wanted to explore what drives the beta diversity amongst different categories (production system) and time. In this regard, we employed the ‘BVSTEP’ routine [24] which reduces the complexity of microbiome datasets by getting rid of any features from the abundance table that do not contribute to beta diversity between samples. Briefly, the procedure calculates pair-wise Bray-Curtis distances between samples using all the features (ASVs). It then records this as a ground truth, and in the second step permutes through the combinations of ASVs, calculates the beta diversity distances over and over again using these permutations, and homes on to minimal subsets of these ASVs where the beta diversity is roughly conserved against the ground truth by maximising the correlation (Supplementary S1). The resulting members of the subset(s) are then tested to see if (a) they still explain the variability between multiple groups (PERMANOVA analysis on these reduced subsets) and (b) if some of the members of these subsets have discriminating abundances between multiple groups (DESeq2, differential heat trees and MINT analysis). By using this approach, we are able to highlight the important members of microbial community. It should be noted that we have employed several analytical techniques to mask out biases associated with their underlying mathematical formulation and to give a consensus agreement on what stands out consistently. The parameterisation of these techniques are as follows: (i) DESeq2 was used with adjusted *p* value significance cut-off of 0.05 and log2 fold change (Supplementary S11), (ii) differential heat tree analysis was performed on proportional normalisation to find clades that were differentially expressed (Fig. 2b) and (iii) MINT analysis was performed to see if we could consolidate the genera that have simultaneous discriminatory power at both spatial (production system N, HW and O) and temporal scales (day 7 and day 30) (Supplementary S2). MINT gives an overall discriminatory power considering all sources of variations including the studies (production system) and temporal scales (days). Whilst MINT gives an overall discriminatory power considering all sources of variations including the studies (production system) and temporal scales (days), it may not be very efficient in terms of picking out the key features, as our results show marked disagreement with DESeq2 and differential heat trees, nonetheless, where it has worked, provided further credence to the underlying patterns being as realistic as they can be. To aid interpretation, all up/downregulated taxa in terms of abundances are annotated with up and down arrows in Figure 1 according to which analysis they were selected in and with taxa from subset analysis as the seeding point.

For production system HW, *Alistipes*, *Ruminococcaceae UCG-014* and *Escherichia-Shigella* were significant genera increasing from day 7 to day 30*. Bacteroides* displayed significant increase at day 7 (decreasing at day 30) using DESeq2 and Heat Tree. For production system O, *Thalassospira*, *Alistipes* and *Bacteroides* increased at day 30 (*Bacteroides* directionality was only observed with MINT analysis). *Lachnoclostridium*, *Eisenbergiella* and *Escherichia-Shigella* all increased at day 7 (decreasing at day 30). For production system N, *Lachnoclostridium* and *Eisenbergiella* increased at day 7 (decreasing at day 30). *Bacteroides* was identified as decreasing at day 7 (increasing at day 30; directionality was only observed with MINT analysis). In general, *Alistipes* was observed to increase consistently at day 30 for all production systems, whilst all other genera showed mixed trends. Although it should be noted that *Alistipes* was present at higher abundance at production system N as compared to others.

Key genera were identified when comparing production systems at day 7. For HW vs O comparison, *Lachnoclostridium* was identified as increased for HW. *Escherichia-Shigella* was identified increased for production system O. For O vs N comparison, *Bacteroides* was increased for O. This was also replicated for HW when comparing to N. Key genera were identified when comparing production systems at day 30. For HW vs O comparison, *Phascolarctobacterium*, *Thalassospira* and *Bacteroides* were identified as increased for O, whereas *Barnesiella* was increased for HW when comparing to O. *Subdoligranulum*, *Eisenbergiella*, *Alistipes*, *Ruminiclostridium* 5 and *Ruminococcaceae UCG-014* were all identified as part of the subsets that explain beta diversity, although they were not implicated as discriminating in differential analyses. For N vs HW comparison at day 30, *Eisenbergiella* was increased for HW, and *Alistipes* was increased for N. For O vs N comparison at day 30, *Bacteroides* was observed with subset analysis alone. *Thalassospira* was observed, yet its discriminatory power was inconclusive.

Core microbiome where genera persist in 85% of the samples (something that is traditionally used to define the prevalence of core microbiome) for different production systems (day 7 and day 30) was assessed (Fig. 2). Genera identified include *Bacteroides*, *Lachnoclostridium*, *Eisenbergiella*, *Ruminoclostridium 9*, *Lactobacillus*, *Ruminococcaceae*, *Shigella flexneri K–671*, *Flavonifractor*, *Ruminococcaceae*, *Lachnospiraceae*, *Ruminiclostridium 5*, *Ruminiclostridium*, *Coprococcus 1* and *Ruminiclostridium 5* at varying level of abundance. In Fig. 2, OTUs are sorted by their abundances with those on the top being low abundant prevalent OTUs, whereas those at the bottom are highly abundant prevalent OTUs.

### Parameters deriving microbial community structure

Analysis of parameters that had a significant effect on microbial diversity were assessed using PERMANOVA against performance parameters when using different dissimilarity measures on microbiome data (Table 1). Using *R*^2^, if significant, to represent the variability explained in the community structure for Bray-Curtis distance, the parameter with the greatest impact was days, explaining 21.2% of the variation. Next, key parameters of interest were *log_CFU_per_g_Campylobacter* (4.1% variability), *Energy_of_Ration* (4.0% variability) and *Protein_perc_ration* (1.2% variability). Using both unweighted and weighted *UniFrac* as a beta diversity measure, the pattern was more or less similar with the same trends with days having the greatest impact on microbial diversity (32.7% and 43.0% respectively). All other parameters had *R*^2^ values at 1–5%.

**Table 1:** Statistics for PERMANOVA against performance parameters when using different dissimilarity measures on microbiome data. *R*^2^ represents the proportion of variability explained, for example, using ‘Days’ and ‘Bray-Curtis’ dissimilarity, the days explain 21.2% variability in microbial community structure

Predictors	Df	*SumsOfSqs*	*MeanSqs*	*F.Model*	*R*^2^	*p*
Bray-Curtis distance						
Days	1	4.9513	4.9513	26.8712	0.21163***	**< 0.001**
No. of parent flocks used	1	1.065	1.065	5.7796	0.04552***	**< 0.001**
Birds placed	1	1.0209	1.0209	5.5407	0.04364***	**< 0.001**
log CFU/g *Campylobacter*	1	0.9606	0.9606	5.2131	0.04106***	**< 0.001**
Protein (% of ration)	1	0.2729	0.2729	1.4813	0.01167^.^	**< 0.1**
Energy of ration (Kcal/lb AME)	1	0.9375	0.9375	5.088	0.04007***	**< 0.001**
Residuals	77	14.1881	0.1843	0.60642		
Total	83	23.3964	1			
Unweighted UniFrac distance						
Days	1	4.2369	4.2369	46.886	0.32727***	**< 0.001**
No. of parent flocks used	1	0.3242	0.3242	3.588	0.02504**	**< 0.01**
Birds placed	1	0.6806	0.6806	7.532	0.05258***	**< 0.001**
log CFU/g *Campylobacter*	1	0.386	0.386	4.272	0.02982***	**< 0.001**
Protein (% of ration)	1	0.1251	0.1251	1.385	0.00966	0.1744
Energy of ration (Kcal/lb AME)	1	0.235	0.235	2.601	0.01816*	0.0165
Residuals	77	6.9582	0.0904	0.53747		
Total	83	12.9461	1			
Weighted UniFrac distance						
Days	1	0.055783	0.055783	76.574	0.43079***	**< 0.001**
No. of parent flocks used	1	0.001894	0.001894	2.6	0.01463^.^	0.0509
Birds placed	1	0.005532	0.005532	7.594	0.04272***	**< 0.001**
log CFU/g *Campylobacter*	1	0.00484	0.00484	6.644	0.03738***	**< 0.001**
Protein (% of ration)	1	0.00092	0.00092	1.262	0.0071	0.2534
Energy of ration (Kcal/lb AME)	1	0.004429	0.004429	6.08	0.0342**	**< 0.01**
Residuals	77	0.056093	0.000728	0.43318		
Total	83	0.129491	1			

^.^*p* < 0.1, *p < 0.05, **p < 0.01, ***p < 0.001

### Direction of influence for extrinsic parameters influencing key metrics for the microbiome

**Fig. 3:**
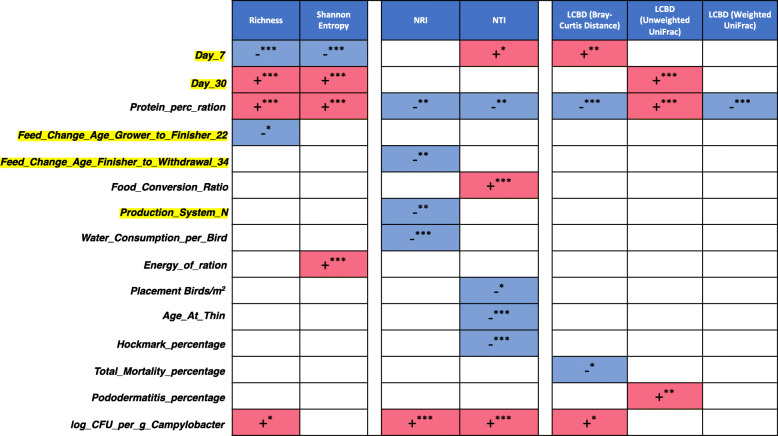
Heatmap of key extrinsic parameters that influence different attributes of microbiome. The figure is based on subset regressions (Supplementary S4 to S10), where red and blue represent the significant positive and negative beta coefficients that were consistently selected in different regression models. The categorical variables are represented with a yellow highlight (coded as 1 (present) or 0 (absent)) and if selected is interpreted as the samples belonging to those categories having positive/negative influence on the respective microbiome metrics

Whilst PERMANOVA analyses show the extent of influence on microbiome structure in terms of variability, to obtain directions as to whether an increase or decrease in these parameters causes an increase or decrease in the properties of microbiome, we resorted to performing subset regressions on one-dimensional realisation of microbiome (alpha, beta diversity measures, etc.). These subset regressions permuted through all possible subsets of explanatory variables (extrinsic parameters considered in this study) by ranking them in terms of quantitative fit after performing cross-validation (Supplementary S4 to S10 and summarised in Fig. 3). Note that red and blue backgrounds represent whether predictors have a positive or a negative influence along with the significances, respectively, in the regression model. In addition, all categorical variables were ‘dummyfied’ (a standard procedure) to represent as present/absent as a tag and were used in the regression model to see whether their inclusion/exclusion has an effect on the final model. As expected, measuring alpha diversity, inclusion of day 30 samples increases richness and Shannon entropy. Although inclusion of day 7 samples led to an increased environmental pressure, it was marginally significant and therefore deterministic nature of microbial communities at local and terminal clade level should be taken with caution. In terms of how samples differ from each other, local contributions to beta diversity (LCBD) was also considered in subset regression analysis with positive/red explanatory variables causing community structure to become different from the average community structure as a mean to identify groups that were markedly different. This was only observed for *Bray-Curtis* distance metric (that considers abundances of taxa without their phylogenetic distances) at day 7, and unweighted *UniFrac* (phylogenetic distance considering only presence/absence of taxa without considering their abundance) for day 30.

An increase in *Protein_perc_ration* led to a positive effect on microbial diversity, whilst simultaneously reducing the effect of environmental pressure on microbial community structure. In terms of beta diversity measure (LCBD with different distances), overall there was a reduction in beta diversity when considering *Protein_perc_ration*, although the trend was opposite for unweighted UniFrac. *Energy_of_ration* also causes the microbial diversity to be more even as it had a positive and significant influence on Shannon entropy. The *Feed_conversion_ratio* on the other hand only caused shift in environmental pressure affecting the terminal clades by making them more clustered though the NTI measure.

In terms of production systems, it was observed that samples belonging to production system N had less influence by the environment and were possibly driven by competitive exclusion principles. Of note, production system N also had the lowest (best) *Feed_conversion_ratio*. A decrease in *Feed_conversion_ratio* was noted to lead to reduced environmental influence, which aligns with production system N having a reduced environmental influence, and also that a higher *Placement_birds/m*^*2*^ resulting in a reduced environmental influence. The same phenomena were also observed when considering *Age_at_thin*, *Hockmark_ percentage* and *Water_consumption _per_bird* as explanatory variables. Interestingly, recorded *log_CFU_per_g_Campylobacter* led to an increase in microbial diversity, with an increase in environmental pressure (at global scale, NRI, and also at local terminal clustering, NTI) as well as causing a marked shift in terms of beta diversity. Of all production systems, *Campylobacter* was only identified in production system N at day 30 based on 16S rRNA abundance count (Supplementary S2), corroborated with independent log CFU/g of *Campylobacter* measure.

## Discussion

Our data clearly show that there is a difference in microbial community structure between production systems with varying influence by extrinsic parameters considered within this study. We observed diversity increase significantly for day 30 when compared to day 7. This is in line with previous reports whereby the gastrointestinal (GI) tract of poultry comes into contact with exogenous microorganisms immediately after hatch and as the host grows, this microbiome becomes highly diverse until it reaches a relatively stable albeit dynamic state [25]. An important finding in this study is that amongst the different production systems, only N is where microbial community assemblage seems to be random (less environmental pressure). Stocking density is clearly a parameter which if varied, can alter microbial community structure significantly. This demonstrates that production systems can be modified to alter the microbiome profile influencing performance at farm level.

Dietary nutrient components are implicated in improving the performance of broiler chickens. An increasing *Energy_of_ration* and decreasing *Protein_perc_ration* in feed over time pertain to all production systems within this study. These variables have a direct influence on gut microbial composition, and in conjunction with differentiating parameters between production systems (i.e. stocking density), lead to differences in microbial composition between production systems. The role of diet is sufficiently important for microbial community structure assemblage as previously described whereby digestion of non-starch polysaccharides (NSPs; found in the grain of chicken feed) lead to production of SCFA, which are absorbed across mucosa and catabolised by the host [15, 26]. SCFAs contribute to chicken nutrition and also lower pH which can inhibit acid-sensitive pathogens and improve mineral absorption [17, 18]. Thus, an association exists between the consistent and differentiating parameters of production systems that affect feed utilisation, leading to competitive exclusion of genera based on competition for nutrients and other factors.

Genera that were differentially expressed between different production systems and days were identified (some also part of the core microbiome). For day 30, HW vs O comparison, *Phascolarctobacterium*, *Thalassospira* and *Bacteroides* were identified as increased for O. *Phascolarctobacterium* is involved in SCFA production, including acetate and propionate and described *as an option for reduction of Campylobacter via competitive exclusion* [27–29]. *Here, Omega-3 fed poultry systems harboured Phascolarctobacterium at a higher prevalence, although we cannot state if this was directly associated with a reduction or absence of Campylobacters from the caecal community. Eisenbergiella* was increased for HW vs N comparison at day 30, and *Alistipes* was increased for N when compared to HW at day 30. A reduction in *Eisenbergiella* has been associated with gastrointestinal disorders linked to metabolic and microbiota changes (functional dyspepsia) resulting in defective energy metabolism, amino acids, nucleotides and SCFA [30]. The HW system may improve the metabolism-microbiome interaction and could result in a competitive exclusion of bacterial pathogens via a fortified immune system. A dysfunctional microbiota can induce metabolic, autoimmune and inflammatory diseases and can seriously undermine gut function [31–33]. *Barnesiella* was increased for HW when comparing to O. Presence of *Barnesiella* has been associated with prevention and spread of highly antibiotic-resistant bacteria [34] where the gut bacterial community may react to antibiotic inclusion in the diet and prophylactically encourage the presence of *Barnesiella*, an effect demonstrated in mice where ampicillin treatment increased their presence [35]. The competitive exclusion hypothesis is supported by the increase of *Alistipes* bacteria in N group at day 30, and using the top-25 most abundant taxa identified at OTU level where clear differences for N against HW and O were observed (also for genera *Bacteroides*). The presence of *Subdoligranulum* bacteria in HW and O production systems, although not discriminatory, still contributing to beta diversity, represents a sign of improved gut health as these bacteria are known to be involved in production of SCFAs (e.g. butyrate) with an important role in gut physiology [36]. *Ruminiclostridium 5*, identified within HW and O production systems, also within core microbiome, has been noted to impact SCFA concentration within the gut [37].

The European Union (EU) ban on antimicrobial growth promoters in 2006 has created an increased need to devise alternative methods to improve performance and potentially reduce numbers of pathogenic bacteria. Examples include use of natural plant-derived products such as carvacrol [38], addition of dietary prebiotics [39] and administration of live probiotic bacteria [40]. More recently, prebiotic galacto-oligosaccharides (GOS) have been added to broiler feed and enhanced the growth rate and feed conversion of chickens relative to those obtained with a calorie-matched control diet [41]. Recent developments have observed chicken diets enriched with Omega-3 PUFA’s for benefits to human health [5]. Omega-3 fatty acids α-linolenic acid (ALA) (often found in plants), eicosapentaenoic acid (EPA) and docosahexaenoic acid (DHA) (found typically in marine oils), all play a role in forming structural components of cell membranes, serve as precursors to bioactive lipid mediators and provide a source of energy [42]. PUFAs are also important as substrates for inflammatory and anti-inflammatory acids, with EPA is believed to have anti-inflammatory properties [43]. One of the proposed mechanisms as to how dietary antibiotics exert their growth promoting benefits is via anti-inflammatory effects towards the intestinal epithelium, by inhibition of production and excretion of catabolic mediators [44]. Increased levels of EPA following antibiotic supplementation align with this non-antibiotic, anti-inflammatory theory of antibiotic growth promotion [45]. In our study, we do not know if PUFAs were fully absorbed, or to what effect the interaction is with host microbiome; however, we do observe an increase in weight for production system O which has Omega-3. Of note, production system N had the lowest (best) FCR.

Remarkably, *log_CFU_per_g_Campylobacter* led to an increase in microbial diversity, an increase in environmental assemblage metrics NRI and NTI, and also increased divergence in community structure from other samples. *Campylobacter* was identified in production system N at day 30 (Supplementary S2) along with measuring the log CFU/g of *Campylobacter* corroborating the 16S data. It has been previously reported that *Campylobacter* typically appears within week two of the life cycle [11–14, 46]. The lack of identification of *Campylobacter* at any of the day 7 samples is anticipated. It is interesting that detection of *Campylobacter* was only observed at day 30 for production system N. This may have been due to limitation of sampling points. Although we recognise that microbial diversity will increase naturally from day 7 to day 30, based on our data, *Campylobacter* is associated with an increasing diversity. Broiler genetics is known to impact microbial diversity [47, 48] and this may explain why in our study the environmental pressure was significantly impacted positively by the presence of *Campylobacter*. Here, the environmental influence may be the chicken host genetics influencing microbial community structure.

## Conclusion

Our findings demonstrate a relative role of different production system parameters in shaping the bacterial communities’ impact on the chicken microbiome, with stocking density playing a major role influencing microbial dynamics. Specific genera with higher prevalence were identified within production systems that have key roles in energy metabolism, amino acid, nucleotide and SCFA utilisation. It is clear that parameters between production systems (whether constant or variable) have an impact on microbial diversity which subsequently influences feed breakdown and hence instigates competitive exclusion of certain genera. Omega-3 had a positive impact on weight gain and *Campylobacter* presence was linked with environmental pressure, which could be either the external environment or the host itself. Future studies that will direct the optimisation of extrinsic parameters and optimisation of diets targeting microbes with the underlying benefits of improving performance will aid in reducing pathogens such as *Campylobacter*. Our study investigates the relative importance of production system parameters in an industrial farm environment without any intervention strategy (studying caecal microbiome at its natural environment), to reveal the factors that link microbial community structure to improved broiler performance and reduced pathogenic bacteria such as *Campylobacter*.

## Supplementary information

**Additional file 1: Supplementary S1-Subset Analysis.docx**: Subset analysis from BVSTEP routine listing top subsets with highest correlation with the full genera table considering Bray-Curtis distance and considering samples provided in the first column. For each subset, PERMANOVA was performed against Groups considered in the first column.

**Additional file 2: Supplementary S2-MINT Analysis.pdf**: MINT study-wise discriminant analysis between treatments (N, HW, and O). Additional methodology provided within file.

**Additional file 3: Supplementary S3-Top 25 Abundant Taxa.pdf**: Community structure based on relative abundance of the top-25 most abundant taxa identified at different taxonomic groups, where ‘others’ refers to all taxa not included in the ‘top-25’.

**Additional file 4: Supplementary S4-Richness Subset Regression.docx**: Subset regression analysis using Richness as the dependent variable.

**Additional file 5: Supplementary S5-Shannon Entropy Subset Regression.docx**: Subset regression analysis using Shannon Entropy as the dependent variable.

**Additional file 6: Supplementary S6-NRI Subset Regression.docx**: Subset regression analysis using phylogenetic alpha diversity (NRI) as the dependent variable.

**Additional file 7: Supplementary S7-NTI Subset Regression.docx:** Subset regression analysis using phylogenetic alpha diversity (NTI) as the dependent variable.

**Additional file 8: Supplementary S8-LCBD Bray-Curtis Subset Regression.docx:** Subset regression analysis using beta diversity measure (LCBD with Bray-Curtis distance) as the dependent variable.

**Additional file 9: Supplementary S9-LCBD Unweighted UniFrac Subset Regression.docx:** Subset regression analysis using beta diversity measure (LCBD with Unweighted UniFrac distance) as the dependent variable.

**Additional file 10: Supplementary S10-LCBD Weighted UniFrac Subset Regression.docx:** Subset regression analysis using beta diversity measure (LCBD with Weighted UniFrac distance) as the dependent variable.

**Additional file 11: Supplementary S11-Pairwise Differential Analysis.xlsx:** Differential analysis of genera that are up/down-regulated between different groups (Adjusted P values ≤0.05) with at least log2 fold change from the base mean abundances for the samples considered in the first column.

**Additional file 12: Supplementary S12-Bioinformatics_and_StatisticalAnalysis.docx:** Bioinformatics and statistical analysis methods used within the study.

**Additional file 13: Supplementary S13-Parameters Measured.docx:** A list of all of the parameters measured within the study and their explanations.

## Data Availability

The raw sequence files supporting the results of this article are available in the European Nucleotide Archive under the project accession number PRJEB34742.

## Abbreviations

N: Normal
HW: Higher Welfare
O: Omega-3 Higher Welfare
SCFA: Short chain fatty acid
FCR: Feed conversion ratio
PUFA: Polyunsaturated fatty acids
AH: As hatched
gDNA: genomic DNA
OTUs: Operational taxonomic units
GI: Gastrointestinal
NSPs: Non-starch polysaccharides
EU: European Union
GOS: Galacto-oligosaccharides
ALA: α-linolenic acid
EPA: Eicosapentaenoic acid
DHA: Docosahexaenoic acid

## Footnotes

1 It should be noted that NRI reflects phylogenetic clustering in a broad sense (whole phylogenetic tree) with lower values representing evenly spread community. On the other hand, NTI focuses more on the tips of the tree with positive values of NTI indicating that species co-occur with more closely related species than expected, and lower values indicating that closely related species do not co-occur by chance
