# Supplementary material for: Impact of industrial production system parameters on chicken microbiomes: mechanisms to improve performance and reduce Campylobacter
Source: Microbiome. 2020 Sep 9;8:128. doi: 10.1186/s40168-020-00908-8 (PMC7488076; doi:10.1186/s40168-020-00908-8)
Supplement: Supplementary file 3 — Additional file 2: Supplementary S2-MINT Analysis.pdf: MINT study-wise discriminant analysis between treatments (N, HW, and O). Additional methodology provided within file. [file 40168_2020_908_MOESM2_ESM.pdf]

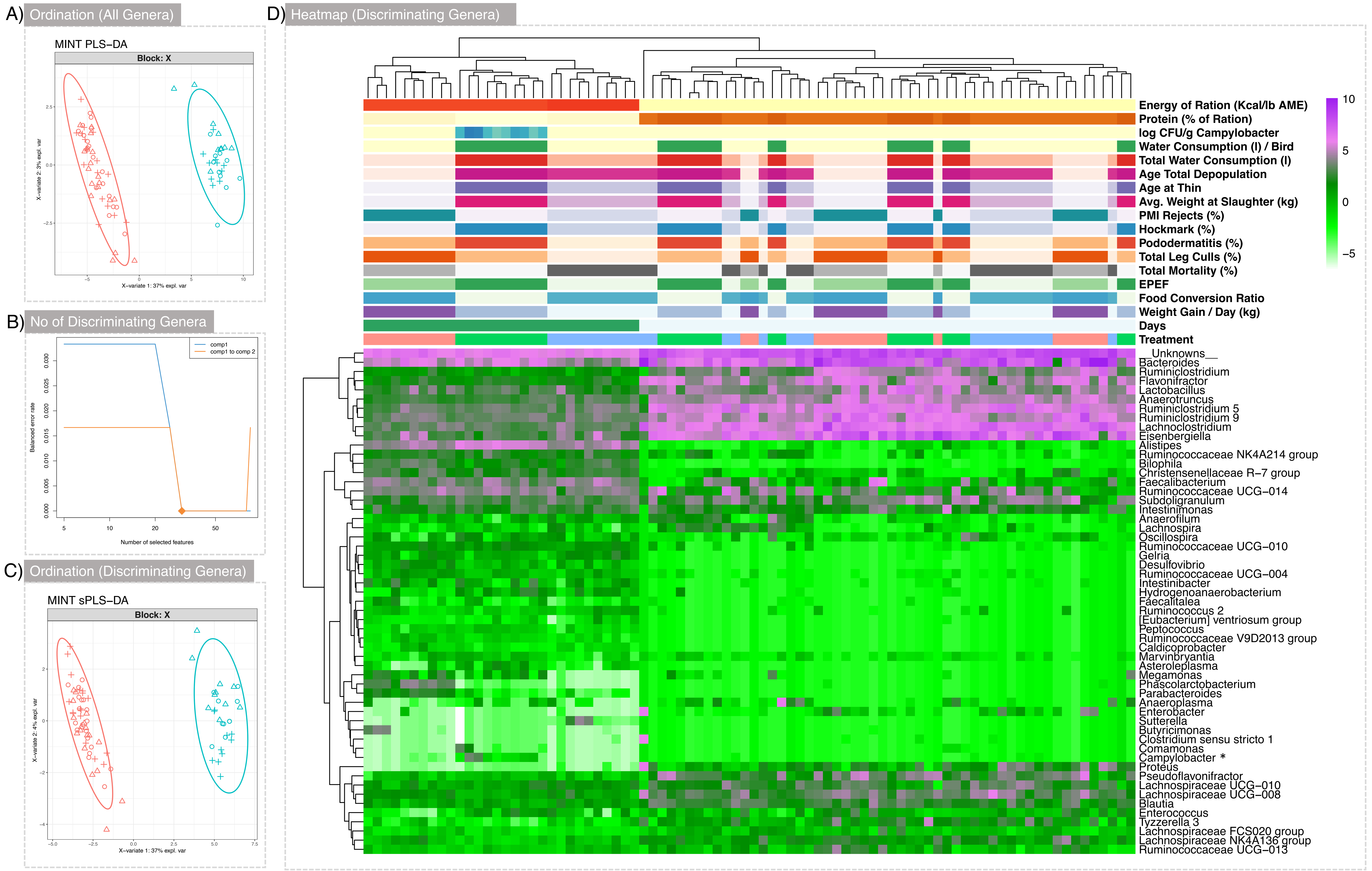

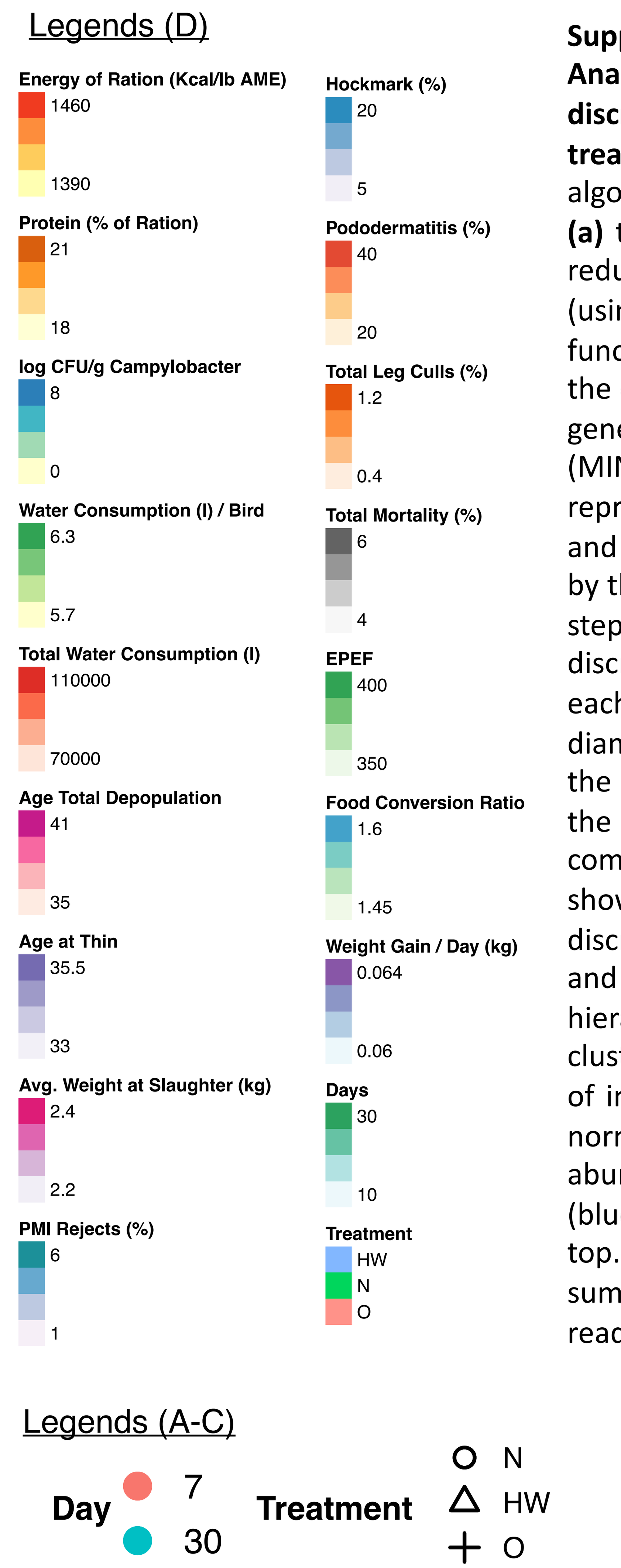

Supplementary S2-MINT Analysis.pdf: **MINT study-wise discriminant analysis between treatments (N, HW, and O)**. The algorithm is a two-step process where **(a)** two components were found that reduce the classification error rates (using mahalanobis.dist in the function) in the algorithm, showing the ordination of samples using all the genera in the first two components (MINT PLS-DA) with ellipse representing 95% confidence interval and percentage variations explained by these components in axes labels. In step two, **(b)** the number of discriminating genera were found for each component, highlighted as diamonds, **(c)** is similar to **(a)** however the ordination was considered using the discriminants from two components (MINT sPLS-DA); **(d)** shows the heatmap of these discriminant genera, with both rows and columns ordered using hierarchical (average linkage) clustering to identify blocks of genera of interest. Heatmap depicts TSS+CLR normalised abundances: high abundance (pink) and low abundance (blue), along with metadata drawn on top. Table on the right side shows the summary statistics for *Campylobacter* reads obtained for each category.

**Campylobacter* Reads in 16S rRNA Samples

	Min	1st. Quar.	Median	Mean	3rd. Quar.	Max
HW 7	0	0	0	0.28	0	2
N 7	0	0	0	0	0	0
O 7	0	0	0	0	0	0
HW 30	0	0	0	0	0	0
N 30	0	13	35	472.50	100.5	2426
O 30	0	0	0	0.5	0.75	3.0
