# Supplementary material for: Impact of industrial production system parameters on chicken microbiomes: mechanisms to improve performance and reduce Campylobacter
Source: Microbiome. 2020 Sep 9;8:128. doi: 10.1186/s40168-020-00908-8 (PMC7488076; doi:10.1186/s40168-020-00908-8)
Supplement: Supplementary file 4 — Additional file 3: Supplementary S3-Top 25 Abundant Taxa.pdf: Community structure based on relative abundance of the top-25 most abundant taxa identified at different taxonomic groups, where ‘others’ refers to all taxa not included in the ‘top-25’. [file 40168_2020_908_MOESM3_ESM.pdf]

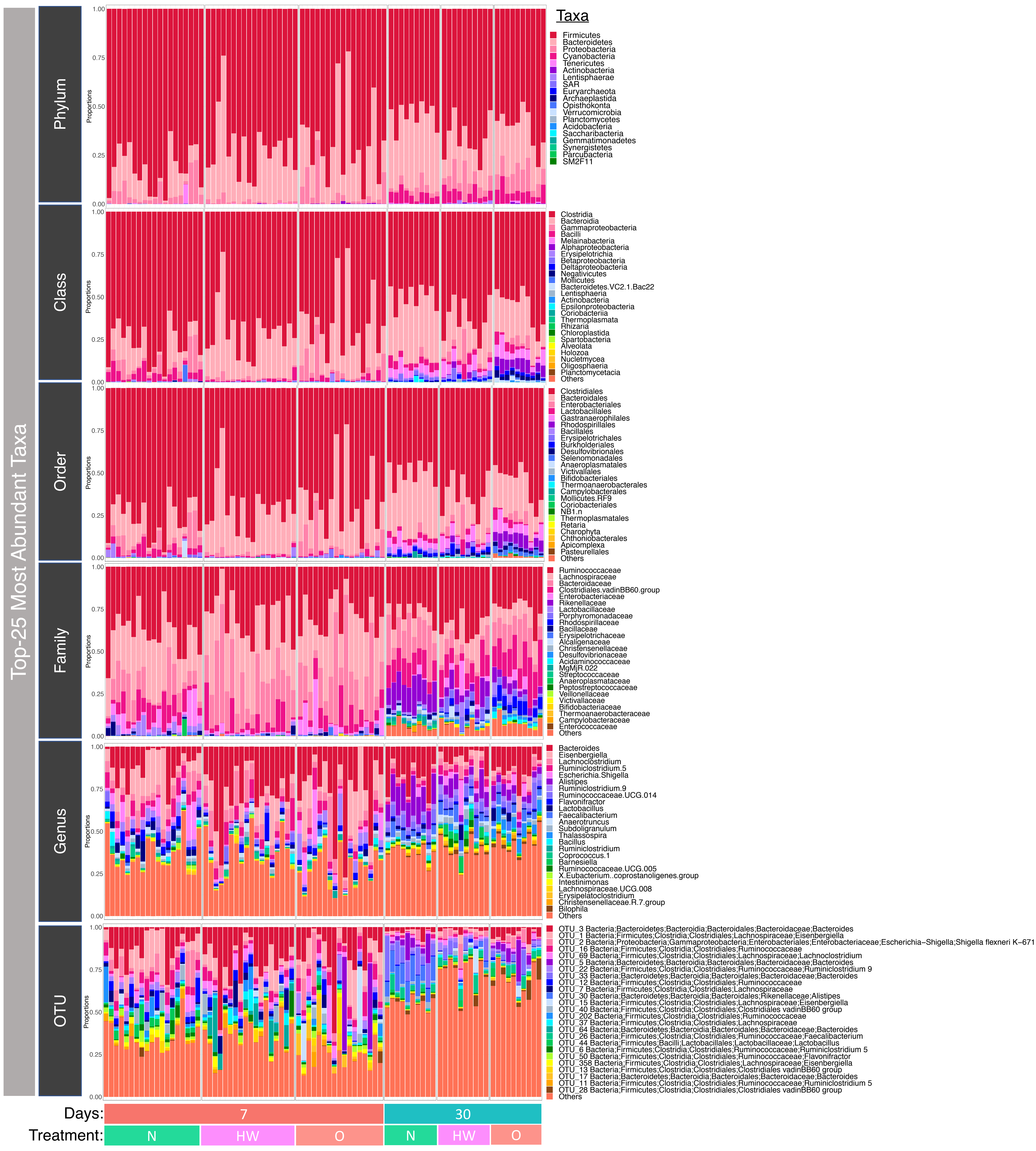
Top-25 Most Abundant Taxa
Phylum
Class
Order
Family
Genus
OTU
Proportions
0.00
0.25
0.50
0.75
1.00
Taxa
Firmicutes
Bacteroidetes
Proteobacteria
Cyanobacteria
Tenericutes
Actinobacteria
Lentisphaerae
SAR
Euryarchaeota
Archaeplastida
Opisthokonta
Verrucomicrobia
Planctomycetes
Acidobacteria
Saccharibacteria
Gemmatimonadetes
Synergistetes
Parcubacteria
SM2F11
Clostridia
Bacteroidia
Gammaproteobacteria
Bacilli
Melainabacteria
Alphaproteobacteria
Erysipelotrichia
Betaproteobacteria
Deltaproteobacteria
Negativicutes
Mollicutes
Bacteroidetes.VC2.1.Bac22
Lentisphaeria
Actinobacteria
Epsilonproteobacteria
Coriobacteriia
Thermoplasmata
Rhizaria
Chloroplastida
Spartobacteria
Alveolata
Holozoa
Nucletmycea
Oligosphaeria
Planctomycetacia
Others
Clostridiales
Bacteroidales
Enterobacteriales
Lactobacillales
Gastranaerophilales
Rhodospirillales
Bacillales
Erysipelotrichales
Burkholderiales
Desulfovibrionales
Selenomonadales
Anaeroplasmatales
Victivallales
Bifidobacteriales
Thermoanaerobacterales
Campylobacterales
Mollicutes.RF9
Coriobacteriales
NB1.n
Thermoplasmatales
Retaria
Charophyta
Chthoniobacterales
Apicomplexa
Pasteurellales
Others
Ruminococcaceae
Lachnospiraceae
Bacteroidaceae
Clostridiales.vadinBB60.group
Enterobacteriaceae
Rikenellaceae
Lactobacillaceae
Porphyromonadaceae
Rhodospirillaceae
Bacillaceae
Erysipelotrichaceae
Alcaligenaceae
Christensenellaceae
Desulfovibrionaceae
Acidaminococcaceae
MgMjR.022
Streptococcaceae
Anaeroplasmataceae
Peptostreptococcaceae
Veillonellaceae
Victivallaceae
Bifidobacteriaceae
Thermoanaerobacteraceae
Campylobacteraceae
Enterococcaceae
Others
Bacteroides
Eisenbergiella
Lachnoclostridium
Ruminiclostridium.5
Escherichia.Shigella
Alistipes
Ruminiclostridium.9
Ruminococcaceae.UCG.014
Flavonifractor
Lactobacillus
Faecalibacterium
Anaerotruncus
Subdoligranulum
Thalassospira
Bacillus
Ruminiclostridium
Coprococcus.1
Barnesiella
Ruminococcaceae.UCG.005
X.Eubacterium..coprostanoligenes.group
Intestinimonas
Lachnospiraceae.UCG.008
Erysipelatoclostridium
Christensenellaceae.R.7.group
Bilophila
Others
OTU_3 Bacteria;Bacteroidetes;Bacteroidia;Bacteroidales;Bacteroidaceae;Bacteroides
OTU_1 Bacteria;Firmicutes;Clostridia;Clostridiales;Lachnospiraceae;Eisenbergiella
OTU_2 Bacteria;Proteobacteria;Gammaproteobacteria;Enterobacteriales;Enterobacteriaceae;Escherichia–Shigella;Shigella flexneri K–671
OTU_16 Bacteria;Firmicutes;Clostridia;Clostridiales;Ruminococcaceae
OTU_69 Bacteria;Firmicutes;Clostridia;Clostridiales;Lachnospiraceae;Lachnoclostridium
OTU_5 Bacteria;Bacteroidetes;Bacteroidia;Bacteroidales;Bacteroidaceae;Bacteroides
OTU_22 Bacteria;Firmicutes;Clostridia;Clostridiales;Ruminococcaceae;Ruminiclostridium 9
OTU_33 Bacteria;Bacteroidetes;Bacteroidia;Bacteroidales;Bacteroidaceae;Bacteroides
OTU_12 Bacteria;Firmicutes;Clostridia;Clostridiales;Ruminococcaceae
OTU_7 Bacteria;Firmicutes;Clostridia;Clostridiales;Lachnospiraceae
OTU_30 Bacteria;Bacteroidetes;Bacteroidia;Bacteroidales;Rikenellaceae;Alistipes
OTU_15 Bacteria;Firmicutes;Clostridia;Clostridiales;Lachnospiraceae;Eisenbergiella
OTU_40 Bacteria;Firmicutes;Clostridia;Clostridiales;Clostridiales vadinBB60 group
OTU_202 Bacteria;Firmicutes;Clostridia;Clostridiales;Ruminococcaceae
OTU_37 Bacteria;Firmicutes;Clostridia;Clostridiales;Lachnospiraceae
OTU_64 Bacteria;Bacteroidetes;Bacteroidia;Bacteroidales;Bacteroidaceae;Bacteroides
OTU_26 Bacteria;Firmicutes;Clostridia;Clostridiales;Ruminococcaceae;Faecalibacterium
OTU_44 Bacteria;Firmicutes;Bacilli;Lactobacillales;Lactobacillaceae;Lactobacillus
OTU_6 Bacteria;Firmicutes;Clostridia;Clostridiales;Ruminococcaceae;Ruminiclostridium 5
OTU_50 Bacteria;Firmicutes;Clostridia;Clostridiales;Ruminococcaceae;Flavonifractor
OTU_358 Bacteria;Firmicutes;Clostridia;Clostridiales;Lachnospiraceae;Eisenbergiella
OTU_13 Bacteria;Firmicutes;Clostridia;Clostridiales;Clostridiales vadinBB60 group
OTU_17 Bacteria;Bacteroidetes;Bacteroidia;Bacteroidales;Bacteroidaceae;Bacteroides
OTU_11 Bacteria;Firmicutes;Clostridia;Clostridiales;Ruminococcaceae;Ruminiclostridium 5
OTU_28 Bacteria;Firmicutes;Clostridia;Clostridiales;Clostridiales vadinBB60 group
Others
Days:
7
30
Treatment:
N
HW
O
N
HW
O
